# 68/w mit progredienter Belastungsdyspnoe und Petechien: Vorbereitung auf die Facharztprüfung: Fall 43

**DOI:** 10.1007/s00108-021-01004-1

**Published:** 2021-04-08

**Authors:** M. Legros, D. Siegrist, U. Bacher

**Affiliations:** 1 Zentrum für Labormedizin, Inselspital, Bern, Schweiz; 2 Universitäre Klinik für Hämatologie, Inselspital, Freiburgstr. 18, 3010 Bern, Schweiz

**Keywords:** Akute myeloische Leukämie, Klassifikation, Therapiekonzepte bei akuten Leukämien, Labordiagnostik, Chemotherapie

## Prüfungssimulation

### Fallschilderung

#### Klinisches Bild.

Eine 68-jährige Frau stellt sich in der Notaufnahme eines Krankenhauses wegen seit ca. 2 Monaten progredienter Belastungsdyspnoe, subfebrilen Temperaturen und rezidivierendem Schüttelfrost sowie neu aufgetretenen Hämatomen an den Extremitäten vor.

#### Untersuchungsbefunde.

- Vitalparameter: Blutdruck 116/48 mm Hg, Puls 100/min, S_p_O_2_ 98 % unter Raumluft, Temperatur 37,6 °C, Atemfrequenz 19–30/min
- 68-jährige Patientin in nur leicht reduziertem Allgemeinzustand und schlankem Ernährungszustand
- Integument: im Bereich der Arme und Beine wenige kleine Hämatome. Im Bereich der Beine nur sehr wenige Petechien
- Enoral: reizlos, im Bereich der Rachenhinterwand lediglich 1–2 Petechien
- Röntgenuntersuchung des Thorax: kardiopulmonal kompensiert, kein Infiltrat

#### Auffällige Laborbefunde.

**Figure 1:**
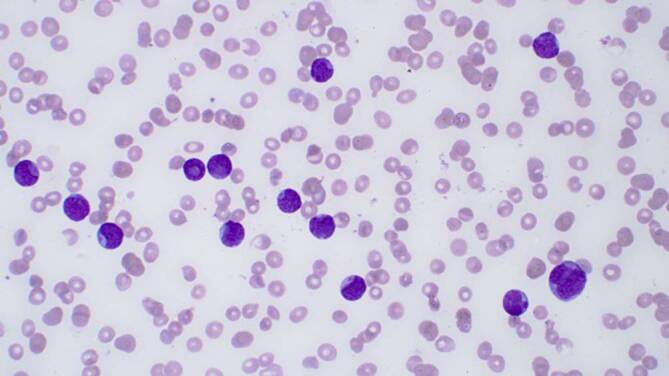

**Figure 2:**
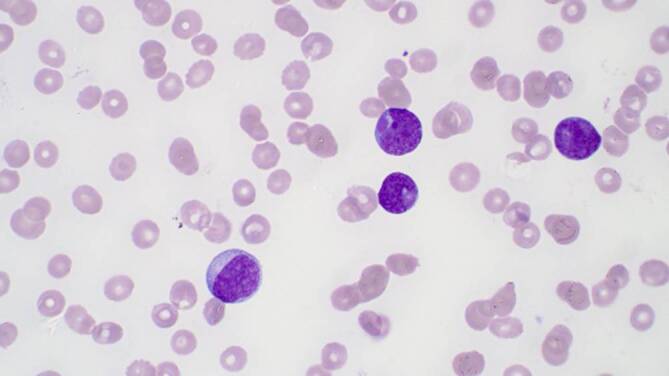

Das Notfalllabor zeigte eine ausgeprägte Anämie (67 g/l) und Thrombozytopenie (36 G/l) sowie eine Leukozytose (60,3 G/l). Im Differenzialblutbild fanden sich 87 % Blasten (Abb. 1 und Abb. 2).

Das Gerinnungslabor war bis auf erhöhte D‑Dimere (8151 µg/l) normal. Die klinisch chemischen Analysen waren bis auf ein erhöhtes C‑reaktives Protein (89 mg/l) und eine erhöhte Laktat-Dehydrogenase (672 IU/l) normal.

## Prüfungsfragen

- Wie lautet Ihre Verdachtsdiagnose?
- Welche weiteren Untersuchungen würden Sie zur Diagnosesicherung verordnen?
- Welche klinischen Symptome treten bei Patienten mit akuter myeloischer Leukämie (AML) auf?
- Welche klinischen Notfallsituationen können bei AML auftreten?
- Nach welchen Klassifikationen werden die AML eingeteilt?
- Nach welcher Klassifikation und zu welchem Zweck erfolgt eine Risikostratifizierung?
- Beschreiben Sie den Ablauf der Erstlinientherapie bei AML.
- Welche weiteren Therapiemöglichkeiten stehen zur Verfügung?

### Antworten

#### Wie lautet Ihre Verdachtsdiagnose?

In Zusammenschau der klinischen Befunde und der Laborergebnisse steht angesichts der Blastenvermehrung im peripheren Blut eine akute Leukämie im Vordergrund. Die relevanten Befunde hierfür sind:

- Leukozyten 60,3 G/l (Referenzbereich 3,0–10,5 G/l)
- 87 % Blasten (unreife Vorläuferzellen) im peripheren Blut
- Hämoglobin 67 g/l (Referenzbereich 121–154 g/l)
- Thrombozyten 36 G/l (Referenzbereich 150–450 G/l)
- Laktat-Dehydrogenase 672 IU/l (Referenzbereich < 250 IU/l)
- Progrediente Dyspnoe
- Hämatome und Petechien

##### Merke.

Eine signifikante Erhöhung von Blasten im peripheren Blut deutet immer auf eine hämatologische Neoplasie hin. Ein Blastenanteil von > 20 % im peripheren Blut erfüllt die Definition einer akuten Leukämie. Bei geringeren Blastenanteilen im peripheren Blut ist der Blastenanteil im Knochenmark entscheidend.

#### Welche weiteren Untersuchungen würden Sie zur Diagnosesicherung verordnen?

Die Diagnose einer akuten Leukämie muss in der Regel mittels einer Knochenmarkuntersuchung bestätigt werden. Das zytomorphologische Bild der Blasten im peripheren Blut liefert oft Hinweise auf die Linienzugehörigkeit (myeloische oder lymphatische Genese), zur definitiven Einteilung und für die Risikostratifizierung sind jedoch ergänzend durchflusszytometrische, zytogenetische und molekulardiagnostische Analysen erforderlich (Tab. 1).

- Zytomorphologische Untersuchung des Knochenmarks
- Immunphänotypisierung mittels Durchflusszytometrie aus peripherem Blut und/oder Knochenmark
- Zytogenetische Untersuchung vorzugsweise aus Knochenmark
- Molekulare Diagnostik aus dem Knochenmark und/oder peripheren Blut

**Table 1:** 

Diagnostische Methode	Interpretation
Visuelle Differenzierung – peripherer Blutausstrich	Blastenanteil, Dysplasien
Zytomorphologische Untersuchung von Knochenmarkaspiraten	Blastenanteil, Dysplasien, Ausreifung; FAB-Klassifikation bei akuter myeloischer Leukämie
Durchflusszytometrie (= multiparametrische Durchflusszytometrie)	Linienzugehörigkeit der akuten Leukämie; Reifegrad einer akuten lymphatischen Leukämie
Histopathologie und Immunhistochemie	Blastenanteil und Linienzugehörigkeit der akuten Leukämie, Zellularität
Zytogenetische Untersuchung (vorzugsweise Knochenmark)	Genetischer Subtyp akuter Leukämien; genetisches Risikoprofil
Molekulardiagnostik (Knochenmark und/oder peripheres Blut) mit PCR-basierten Methoden und/oder Next Generation Sequencing	Genetischer Subtyp akuter Leukämien; genetisches Risikoprofil

*FAB* French-American-British, *PCR* Polymerase-Kettenreaktion

#### Welche klinischen Symptome treten bei Patienten mit akuter myeloischer Leukämie auf?

**Figure 3:**
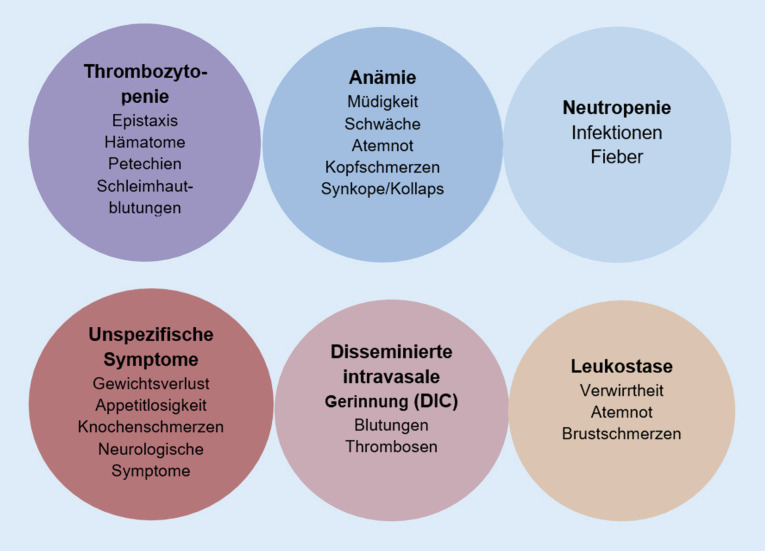

Die Symptome bei AML (Abb. 3) sind meist unspezifisch und mannigfaltig, sie sind bedingt durch:

- Zytopenien
- Gerinnungsstörungen, ggf. als disseminierte intravasale Gerinnung (DIC). Die Kontrolle des kompletten Gerinnungsstatus ist deshalb obligatorisch.

Die AML kann sich auch extramedullär manifestieren (z. B. Gingivahyperplasie; kutane Infiltrationen; Chlorome). Bei Befall des Zentralnervensystems kann eine Meningeosis leucaemica resultieren.

#### Welche klinischen Notfallsituationen können bei akuter myeloischer Leukämie auftreten?

**Figure 4:**
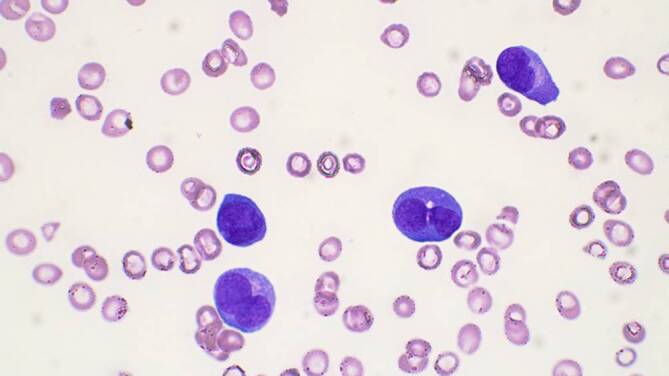

In der Regel ist es vertretbar, den Therapiebeginn um wenige Tage zu verzögern, bis die Resultate der gesamten Diagnostik vorliegen. In folgenden Fällen ist eine unmittelbare Intervention unabdingbar:

- **Hyperleukozytose und Leukostase**
  - Die Hyperleukozytose beschreibt eine leukämiebedingte ausgeprägte Leukozytose mit Werten > 100 G/l.
  - Als Leukostase wird ein sekundäres Hyperviskositätssyndrom aufgrund einer Hyperleukozytose bezeichnet. Sie wird hauptsächlich im Rahmen monozytär differenzierter Subtypen der AML beobachtet.
  - Die Frühmortalität innerhalb der ersten Woche liegt bei 20–40 %. Eine direkte Korrelation zwischen Zellzahl und Mortalitätsrate ist nicht festzustellen, doch haben Patienten mit respiratorischen oder neurologischen Symptomen eine besonders ungünstige Prognose.
    Diese Patienten bedürfen einer sofortigen internistischen Intervention (oftmals sind intensivmedizinische Maßnahmen notwendig): forcierte Hydratation, Tumorlyseprophylaxe bzw. -therapie, mitunter Nierenersatzverfahren. Zytoreduktive Maßnahmen mittels Chemotherapie und ggf. Leukapherese sind ebenso essenziell.
- **Akute Promyelozytenleukämie (APL)**
  - Die APL ist ein seltenerer Subtyp der AML.
  - Die vorläufige Diagnose einer APL ergibt sich in vielen Fällen aus der Untersuchung des peripheren Blutausstrichs (Abb. 4 und 5), in dem sich typischerweise blastäre Zellen mit zahlreichen Auer-Stäbchen, sogenannte Faggot-Zellen, finden.
  - Die APL zeichnet sich durch eine reziproke Translokation zwischen den Chromosomen 15 und 17 aus. Dies führt zur Fusion der Gene *PML* (promyelozytische Leukämie) und *RARA* (Retinsäurerezeptor α).
  - Gefürchtet bei APL sind schwere Blutungen aufgrund von Gerinnungsstörungen (DIC, Fibrinolyse), oftmals mit vitaler Gefährdung und dem Risiko der Frühmortalität.
  - Sobald die Diagnose einer APL vermutet wird, sollte eine Therapie mit All-trans-Retinsäure (ATRA), evtl. kombiniert mit Arsentrioxid, gestartet werden – noch vor der weiteren Diagnosesicherung durch genetische Verfahren. Diese Therapie vermag die Gerinnungsstörungen, die häufig mit APL in Verbindung gebracht werden, zu verhindern oder zu begrenzen.
  - Bei der Behandlung von Patienten mit APL muss auf die Entstehung eines sog. ATRA-Differenzierungssyndroms geachtet werden (unerklärliches Fieber, Gewichtszunahme, Dyspnoe mit Lungeninfiltraten, Pleura- und Perikarderguss, Hypotonie und Nierenversagen). Die Pathogenese des ATRA-Differenzierungssyndroms ist nur unvollständig bekannt, scheint aber mit der großen Menge ausreifender myeloischer Zellen und deren Produktion von inflammatorischen Zytokinen verbunden zu sein. Somit gilt eine hohe Tumorlast (angezeigt durch hohe periphere Leukozyten) als Risikofaktor. Oft werden Kortikosteroide zur Behandlung des Differenzierungssyndroms eingesetzt.

**Figure 5:**
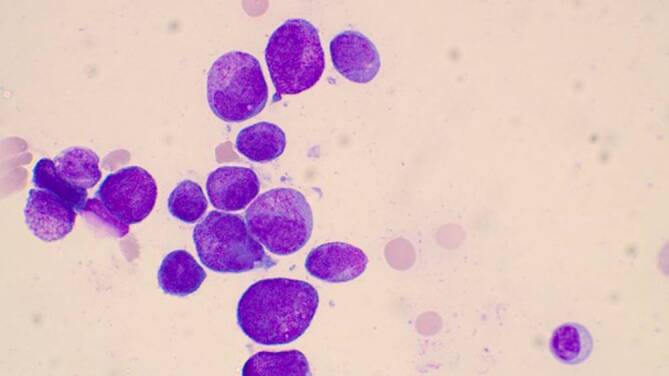

#### Nach welchen Klassifikationen werden die akuten myeloischen Leukämien eingeteilt?

- Die aus den 1970er-Jahren stammende **FAB-Klassifikation** (Tab. 2) ist ein System zur zytomorphologischen Einteilung von AML, akuten lymphatischen Leukämien (ALL) und myelodysplastischen Syndromen (MDS).
- Während die damaligen Klassifikationen für die ALL und das MDS im diagnostischen Alltag inzwischen weniger Anwendung finden, werden die AML-Fälle nach wie vor gemäß FAB-Klassifikation eingeteilt [1].
- Die Klassifikation der Weltgesundheitsorganisation (WHO; Tab. 3) für akute myeloische Leukämien bezieht neben phänotypischen Aspekten auch genetische Veränderungen ein [2].

**Table 2:** 

*M0*	Minimal differenzierte AML
*M1*	Akute Myeloblastenleukämie
*M2*	Akute Myeloblastenleukämie mit Ausreifung
*M3*	APL
*M3v*	Variante der APL
*M4*	Akute myelomonozytäre Leukämie
*M4Eo*	Akute myelomonozytäre Leukämie mit Eosinophilie
*M5*	M5a: akute Monoblastenleukämie M5b: akute Monozytenleukämie
*M6*	Erythroleukämie
*M7*	Akute Megakaryoblastenleukämie

*AML* akute myeloische Leukämie, *APL* akute Promyelozytenleukämie, *FAB* French-American-British

**Table 3:** 

*AML mit rekurrenten genetischen Anomalien*
– AML mit t(8;21)(q22;q22.1); *RUNX1-RUNX1T1*
– AML mit inv(16)(p13.1q22) oder t(16;16)(p13.1;q22); *CBF-MYH11*
– Akute Promyelozytenleukämie mit *PML-RARA*
– AML mit t(9;11)(p21.3;q23.3); *MLLT3-KMT2A*
*AML mit myelodysplasieassoziierten Veränderungen*
*Therapieassoziierte myeloische Neoplasie*
*AML, nicht anderweitig klassifiziert („not otherwise specified“ [NOS])*
– FAB-Klassifikation
– Akute Basophilenleukämie
– Akute Panmyelose mit Myelofibrose
*Myeloisches Sarkom*
*Myeloische Down-Syndrom-assoziierte Proliferation*

*AML* akute myeloische Leukämie, *FAB* French-American-British, *inv* Inversion, *t* Translokation

#### Nach welcher Klassifikation und zu welchem Zweck erfolgt eine Risikostratifizierung?

- Eine Expertengruppe des European LeukemiaNet (ELN) hat zur besseren Vergleichbarkeit klinischer Studiendaten eine Risikostratifizierung für Patienten mit AML erarbeitet (Tab. 4), die auf zyto- und molekulargenetischen Charakteristika beruht. Diese unterscheidet drei Risikogruppen: günstig, intermediär, ungünstig [3].
- Weiter müssen patientenspezifische Faktoren wie Alter, Komorbiditäten und vorangegangene Radio- oder Chemotherapien sowie die Leukozytenzahl bei Diagnose und Manifestation der AML (medullär/extramedullär) berücksichtigt werden.
- Die Risikostratifizierung ist für die Wahl der Therapie relevant.

**Table 4:** 

Risikogruppe	Genetische Veränderung
**Günstig**	t(8;21)(q22;q22); *RUNX1-RUNX1T1*
	inv(16)(p13.1q22) oder t(16;16)(p13.1;q22); *CBFB-MYH11*
	*NPM1*-Mutation ohne *FLT3*-ITD (normaler Karyotyp)
	*CEBPA*-Mutation (normaler Karyotyp)
**Intermediär‑I**	*NPM1*-Mutation und *FLT3*-ITD (normaler Karyotyp)
	*NPM1*-Wildtyp und *FLT3*-ITD (normaler Karyotyp)
	*NPM1*-Wildtyp ohne *FLT3*-ITD (normaler Karyotyp)
**Intermediär-II**	t(9;11)(p22;q23); *MLLT3-MLL*
	Zytogenetische Veränderungen, die nicht als günstig oder ungünstig eingeteilt werden
**Ungünstig**	inv(3)(q21q26.2) oder t(3;3)(q21;q26.2); *RPN1-EVI1*
	t(6;9)(p23;q34); *DEK-NUP214*
	t(v;11)(v;q23); *MLL* (*KMT2A*) rearrangiert
	−5 oder del(5q);−7; abnl(17p); komplexer Karyotyp

*abnl* Anomalie, *del* Deletion, *inv* Inversion, *ITD* interne Tandemduplikation, *t* Translokation

#### Beschreiben Sie den Ablauf der Erstlinientherapie bei akuter myeloischer Leukämie

- Die Therapie akuter Leukämien erfolgt in mehreren Phasen. Begonnen wird bei AML-Patienten, bei denen ein kurativer Ansatz verfolgt wird bzw. verfolgt werden kann, mit einer intensiven Induktionschemotherapie. Die meisten Induktionsschemata sehen 2 Induktionszyklen vor. Es werden Anthrazykline und Cytarabin kombiniert. Häufig wird das sogenannte 7 + 3-Schema eingesetzt.
- Ziel der Induktionstherapie ist die komplette Remission, also die vollständige Erholung des Blutbilds und eine Minimierung der leukämischen Zellen im Knochenmark.
- Im Anschluss an die Induktionstherapie erfolgt die Konsolidierungstherapie. Diese variiert je nach Risikoeinteilung der Erkrankung. Patienten mit günstigerem Risikoprofil werden mit einer erneuten intensiven Chemotherapie oder autologen Stammzelltransplantation behandelt, Patienten mit ungünstigem Risikoprofil sind Kandidaten für eine allogene Stammzelltransplantation.
- Bei Patienten, die aufgrund eines eingeschränkten Allgemeinzustands oder umfangreicher Komorbiditäten nicht für eine intensive Therapie infrage kommen, werden palliative Therapieregime eingesetzt, oftmals mit hypomethylierenden Substanzen (Azacitidin oder Decitabin). Diese Regime können temporär die Krankheit stabilisieren und das Überleben zumindest verbessern [5].

#### Welche weiteren Therapiemöglichkeiten stehen zur Verfügung?

- **FLT3** ist eine Rezeptortyrosinkinase. Die *FLT3*-ITD-Mutation ist eine der am häufigsten nachweisbaren Mutationen bei AML. Diese Mutation führt zur verstärkten Aktivierung der betreffenden Rezeptortyrosinkinase und verstärkten Zellproliferation. Sie ist prognostisch ungünstig.
- FLT3 kann durch Tyrosinkinaseinhibitoren (TKI) gehemmt werden. Die entsprechende Wirkstoffgruppe bezeichnet man als FLT3-Inhibitoren. Zu den Substanzen dieser Gruppe gehören u. a. Midostaurin und Gilteritinib. Man spricht auch von *„targeted therapies“*.
- Weitere Beispiele für neue „targeted therapies“ bei AML sind IDH1- oder IDH2-Inhibitoren für Patienten mit den entsprechenden Mutationen.
